# Effect of Marine-Derived Saccharides on Human Skin Fibroblasts and Dermal Papilla Cells

**DOI:** 10.3390/md21060330

**Published:** 2023-05-27

**Authors:** Aleksandra Augustyniak, Helena McMahon

**Affiliations:** Circular Bioeconomy Research Group, Shannon Applied Biotechnology Centre, Munster Technological University-Kerry, Clash, V92CX88 Tralee, Co. Kerry, Ireland; aleksandra.augustyniak@mtu.ie

**Keywords:** dermal fibroblasts, dermal papilla cells, L-fucose, chondroitin sulphate disaccharide, proliferation, extracellular matrix

## Abstract

The skin is the largest organ of the human body, composed of a diverse range of cell types, non-cellular components, and an extracellular matrix. With aging, molecules that are part of the extracellular matrix undergo qualitative and quantitative changes and the effects, such as a loss of skin firmness or wrinkles, can be visible. The changes caused by the aging process do not only affect the surface of the skin, but also extend to skin appendages such as hair follicles. In the present study, the ability of marine-derived saccharides, L-fucose and chondroitin sulphate disaccharide, to support skin and hair health and minimize the effects of intrinsic and extrinsic aging was investigated. The potential of the tested samples to prevent adverse changes in the skin and hair through stimulation of natural processes, cellular proliferation, and production of extracellular matrix components collagen, elastin, or glycosaminoglycans was investigated. The tested compounds, L-fucose and chondroitin sulphate disaccharide, supported skin and hair health, especially in terms of anti-aging effects. The obtained results indicate that both ingredients support and promote the proliferation of dermal fibroblasts and dermal papilla cells, provide cells with a supply of sulphated disaccharide GAG building blocks, increase ECM molecule production (collagen and elastin) by HDFa, and support the growth phase of the hair cycle (anagen).

## 1. Introduction

The skin is the largest organ in the body, consisting of many cellular layers and a diverse array of cells. As the human body ages, the quantity and quality of key skin building molecules, such as collagen, elastin, and proteoglycans, decreases; the composition of the extracellular matrix is altered; and the skin becomes thinner and less resistant to environmental conditions [1]. Numerous changes taking place at the cellular and molecular level also become visible on the skin surface as wrinkles or skin laxity. The aging process occurs as a result of external and internal factors, for example, genetic, hormonal, chronological, and environmental factors, sun exposure, dietary habits, and lifestyle. Intrinsic aging can be characterized by three main features: atrophy of the dermis due to loss of collagen, degeneration in the elastic fiber network, and loss of hydration. Extrinsic aging is often referred to as photoaging, as it is primarily caused by ultraviolet (UV) exposure. The distinguishing feature of photoaging is an accumulation of elastotic material in the upper and middle dermis (solar elastosis) [2]. The progressive impact of aging is particularly visible in the skin. The proportion of activated fibroblasts in the dermis changes and the density of the extracellular matrix decreases. The integrity of the skin is disturbed, skin becomes thinner, and wound healing and regenerative abilities are compromised [3,4]. The changes caused by the aging process do not only affect the surface of the skin, but also extend to skin appendages, such as hair follicles [5]. Progressive hair loss and reduced regeneration capacity of the hair follicle was observed in humans and rodents [6,7,8]. Regeneration of hair follicles is orchestrated by epithelial–mesenchymal interactions between stem/progenitor cells and fibroblasts comprising the dermal papilla [9,10]. Dermal papilla cells are responsible for controlling proliferation and differentiation of epithelial progenitors through releasing a cascade of signaling molecules [11]. Age-related hair loss (sometimes referred to as senescent alopecia) is associated with a miniaturization of hair follicles and a reduction in dermal papilla cell numbers [12].

**Figure 1:**
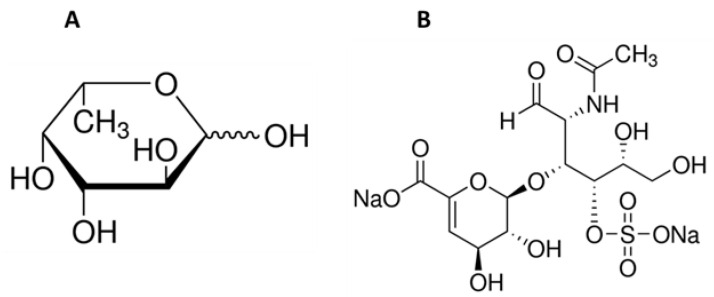
Chemical structure of L-fucose (**A**) and chondroitin disaccharide ∆di-4S sodium salt (**B**).

Water, a source of life on Earth and essential for all living organisms, also represents the origin of biologically active chemicals which are incredibly valuable for the pharmaceutical industry. Marine organisms are a source of many ingredients which have various useful roles in maintaining health. They can be applied in the fight against reactive oxygen species (ROS) [13,14,15,16,17] and in the treatment or prevention of infectious diseases [18,19,20,21], cancer diseases [22,23,24,25,26], diabetes [27,28], and obesity [29,30,31], as well as cardiovascular illnesses caused by hypertension [32,33]. Marine-derived compounds are used to produce drugs, pharmaceuticals, and supplements, as well as cosmetics. Various bioactive extracts from marine plants and animals are used in cosmetics acting as photo-aging inhibitors [34,35,36], skin whitening agents [37,38,39], or antiacne agents [40]. Some of these valuable ingredients are not found outside the marine environment. An example of such a group of compounds might be fucose-containing sulfated polysaccharides, with the best-known representative fucoidan. Fucoidans can be characterized by variability in chemical structure, dependent on the source, season, or plant maturity, but L-fucose (Figure 1A) is always the main component and may exceed 90% of the total fucoidan sugar composition [41]. Marine sulphated polysaccharide naturally occur in brown algae (*Phaeophyta*) and seawater invertebrates such as echinoderms (sea urchins and cucumbers) (class *Echinoidea* and *Holothuroidea*). The sea cucumber is also a source of another compound that occurs exclusively in the marine environment—fucosylated chondroitin sulfate [42]. This compound belongs to the glycosaminoglycan group and has the same backbone as mammalian chondroitin sulfate (D-glucuronic acid and N-acetyl-D-galactosamine; Figure 1B) but is distinguished by the presence of sulfated L-fucose [43]. This unusual sugar, characterized by the lack of a hydroxyl group on the 6-position carbon and the L-configuration, occurs in nature in tiny amounts and can be found in humans, bacteria, plants, and fungi [44,45]. L-fucose is a major component of human milk oligosaccharides and can also be found in mucin glycoproteins and glycolipids [44,46,47].

Fucoidans extracted from many marine sources, as well as glycosaminoglycans, are known for their bioactivities, including their effects on the skin and hair. Fucoidans obtained from various marine macroalgae promote the proliferation of dermal fibroblasts and dermal papilla cells, stimulate hair growth in vitro and in vivo, and have anti-aging properties [48,49,50,51,52,53,54,55]. Glycosaminoglycans, including chondroitin sulphate, have been proven to be skin anti-ageing ingredients/agents/compounds, wound healing promoters, and antiapoptotic agents [56,57,58,59].

In the present study, the effect of single compounds, the basic units of fucoidan and chondroitin sulphate, L-fucose and chondroitin disaccharide ∆di-4S sodium salt, on skin- and hair-derived cells was investigated. We aimed to understand the bioactivity of tested samples on the cellular level and to assess if they can be considered a potential treatment for skin and hair ageing. The potential ability of L-fucose and chondroitin disaccharide to prevent adverse changes in the skin and hair caused by ageing processes was analyzed. Commonly known hair growth markers, such as cell proliferation and the production of alkaline phosphatase and glycosaminoglycans, were tested. In order to look deeper into the hair-related bioactivity of the tested compounds, an analysis of hair-cycle-related genes by qRT-PCR was performed. The influence of the tested samples on skin health was investigated by performing a proliferation assay, an assessment of the extracellular matrix composition, and quantification of type I collagen and elastin. To the knowledge of the authors, the data obtained in the present study are the first of its kind. The bioactivities of L-fucose and chondroitin disaccharide ∆di-4S sodium salt, the building units of larger molecules with proven biological activities, have not been investigated previously in the skin and hair cellular models used in our study.

## 2. Results

### 2.1. Effect of L-fucose and Chondroitin Disaccharide ∆di-4S Sodium Salt on Dermal Fibroblast Proliferation Rate

The proliferation rate of fibroblasts in the dermis slows down through aging and a decrease in the total number of dermal cells is observed. To determine whether the tested samples have the ability to affect the proliferation rate of human dermal fibroblasts, PrestoBlue™ Cell Viability Reagent was used.

**Figure 2:**
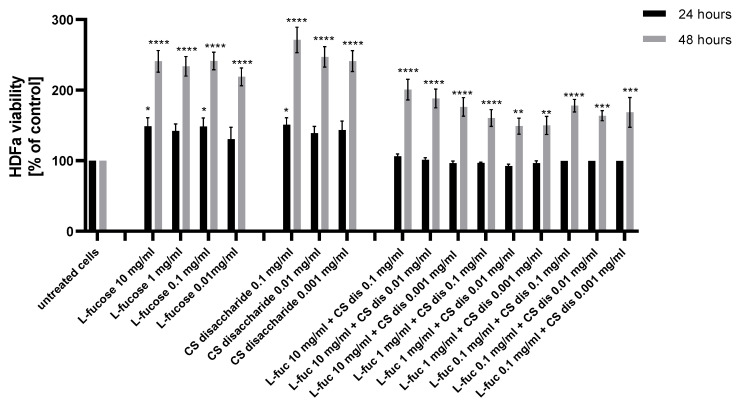
Effect of L-fucose and chondroitin disaccharide Δdi-4S sodium salt on cell proliferation of human dermal fibroblasts. The data are presented as the means ± SEM. Statistical significance in comparison to the negative control (untreated cells) was assessed using a one-way ANOVA followed by Dunnett’s multiple comparison test; * *p* < 0.05; ** *p* < 0.01; *** *p* < 0.001; **** *p* < 0.0001.

As can be seen from Figure 2, human dermal fibroblast exposure to L-fucose and chondroitin sulphate disaccharide resulted in a statistically significant increase in the cellular proliferation rate.

The effect was observed for all tested samples after 48 h of incubation. None of the tested samples were cytotoxic against used cell lines, with the lowest value of 92.5% obtained after 24 h treatment with a combination of 1 mg/mL L-fucose and 0.01 mg/mL chondroitin sulphate disaccharide. Two samples with concentrations of L-fucose of 10 mg/mL and 0.1 mg/mL and of chondroitin sulphate disaccharide of 0.1 mg/mL and 0.001 mg/mL resulted in a statistically significant increase in HDFa proliferation after 24 h incubation. Chondroitin sulphate disaccharide sodium salt has the strongest proliferative effect on human dermal fibroblasts. The effect of separate treatment of dermal fibroblasts with tested L-fucose and chondroitin sulphate disaccharide in terms of the obtained proliferation values was significantly better in comparison to the cell’s exposure to treatment with the samples combined.

### 2.2. Effect of L-fucose and Chondroitin Disaccharide ∆di-4S Sodium Salt on Dermal Papilla Cell Proliferation Rate

To examine whether the tested L-fucose and chondroitin sulphate disaccharide have the ability to affect the growth phase of the hair cycle (anagen), the proliferation rate of human dermal fibroblasts was investigated by using PrestoBlue™ Cell Viability Reagent.

**Figure 3:**
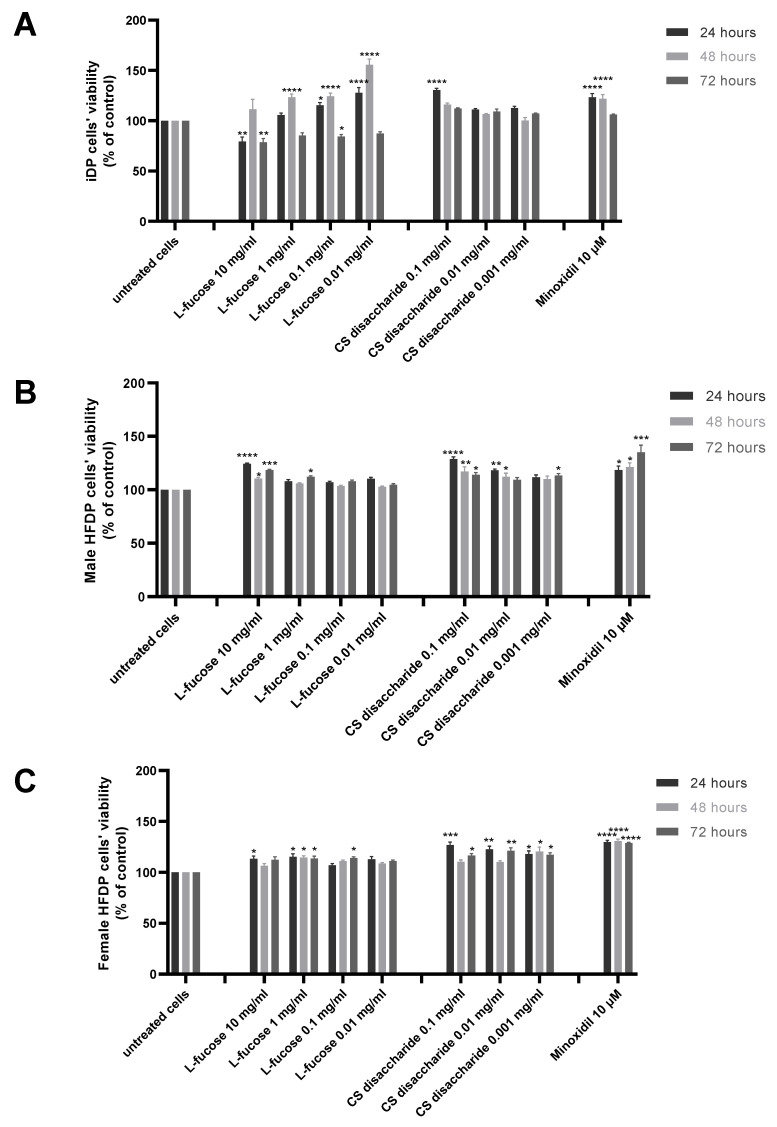
Effect of L-fucose and chondroitin disaccharide Δdi-4S sodium salt on cell proliferation of dermal papilla cells: immortalized (**A**), primary male (**B**), and primary female (**C**). Cells were treated with different concentrations of the tested samples for 24, 48, and 72 h, and proliferation was assessed using PrestoBlue^®^ Cell Viability Reagent. The data are presented as the means ± SEM. Statistical significance in comparison to the negative control (untreated cells) was assessed using a one-way ANOVA followed by Dunnett’s multiple comparison test; * *p* < 0.05; ** *p* < 0.01; *** *p* < 0.001; **** *p* < 0.0001.

The results of experiments performed on three types of dermal papilla cells are presented in Figure 3. Two tested concentrations of L-fucose, 10 mg/mL and 0.1 mg/mL, caused a statistically significant decrease in the proliferation of immortalized dermal papilla cells (Figure 2A); however, the obtained results do not demonstrate a cytotoxic effect of the tested sample. In the case of primary cell lines (Figure 2B,C), the tested L-fucose and chondroitin disaccharide Δdi-4S sodium salt significantly stimulated the proliferation of male- and female-derived dermal papilla cells. A statistically significant increase in hair cell proliferation in comparison to the untreated cells was also observed for 10 µM minoxidil.

**Figure 4:**
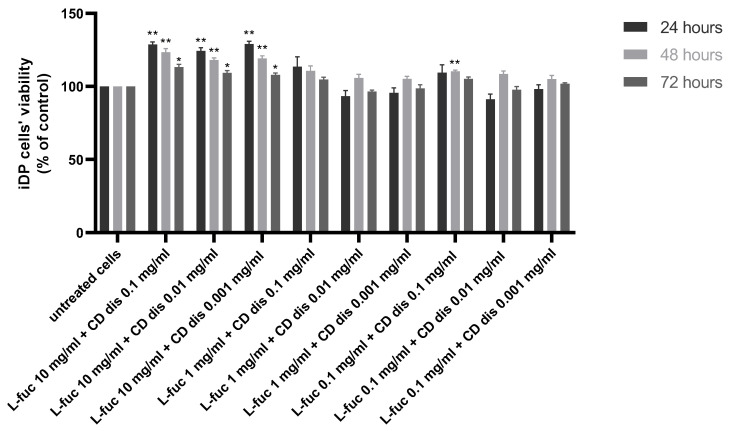
Effect of the simultaneous treatment of immortalized dermal papilla cells with L-fucose and chondroitin disaccharide Δdi-4S sodium salt. Cells were treated with different concentrations of tested samples for 24, 48, and 72 h, and proliferation was assessed using PrestoBlue^®^ Cell Viability Reagent. The data are presented as the means ± SEM. Statistical significance in comparison to the negative control (untreated cells) was assessed using a one-way ANOVA followed by Dunnett’s multiple comparison test; * *p* < 0.05; ** *p* < 0.01.

The combination of 10 mg/mL L-fucose with the three different concentrations of chondroitin sulphate disaccharide caused a statistically significant increase in immortalized dermal papilla cell proliferation at all tested timepoints (Figure 4).

### 2.3. Effect of L-fucose and Chondroitin Disaccharide ∆di-4S Sodium Salt on Alkaline Phosphatase Levels in Dermal Papilla Cells

To investigate whether the tested samples influence the anagen phase of hair growth, the level of alkaline phosphatase production by immortalized and primary dermal papilla cells was assessed.

**Figure 5:**
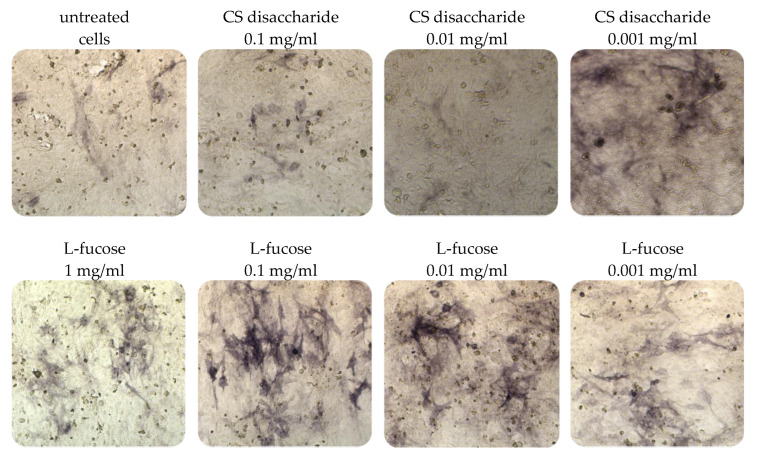
Effect of L-fucose and chondroitin disaccharide ∆di-4S sodium salt on alkaline phosphatase production by immortalized dermal papilla cells. Cells were treated with different concentrations of the tested samples for 120 h and stained with 5-bromo-4-chloro-3-indolyl phosphate/nitroblue tetrazolium (BCIP/NBT) to visualize alkaline phosphatase activity (bluish violet color).

Immortalized dermal papilla cells cultured for 120 h in media containing L-fucose or chondroitin sulphate disaccharide sodium salt displayed alkaline phosphatase activities (Figure 5). The results of the histological analysis showed an increase in the enzyme activity (bluish violet color). The highest intensity of staining in comparison to untreated cells was observed when dermal papilla cells were cultured with two concentrations of L-fucose, 0.1 mg/mL and 0.01 mg/mL. Fewer violet cells were noted in the chondroitin sulphate disaccharide-treated group, especially at a concentration of 0.001 mg/mL.

**Figure 6:**
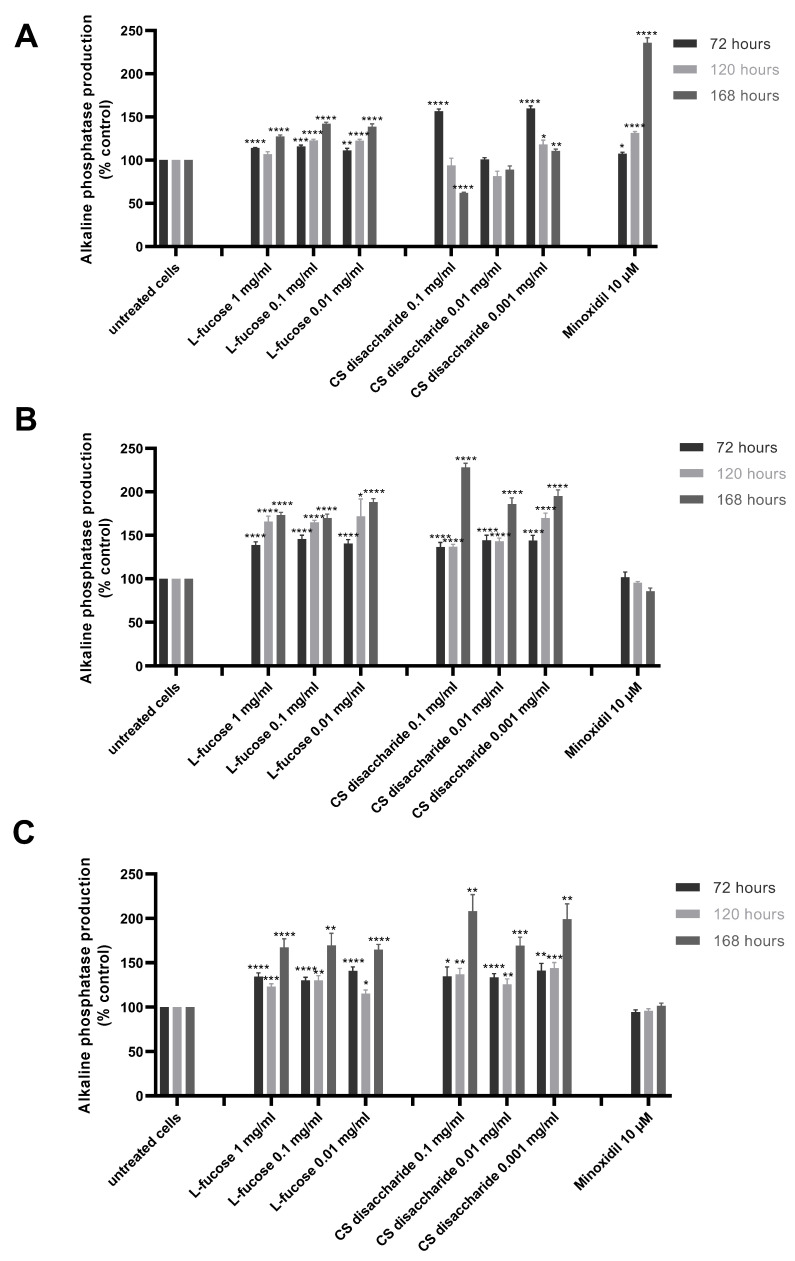
Effect of L-fucose and chondroitin disaccharide Δdi-4S sodium salt on alkaline phosphatase production by dermal papilla cells: immortalized (**A**), primary male (**B**), and primary female (**C**). Cells were treated with different concentrations of tested samples for 72, 120, and 168 h. The data are presented as the means ± SEM. Statistical significance in comparison to the negative control (untreated cells) was assessed using a one-way ANOVA followed by Dunnett’s multiple comparison test; * *p* < 0.05; ** *p* < 0.01; *** *p* < 0.001; **** *p* < 0.0001.

The results from the quantitative analysis of enzyme production indicated that tested samples significantly increase alkaline phosphatase expression, this was especially visible in the case of L-fucose used at the three sampled concentrations and chondroitin sulphate disaccharide at the lowest concentration, 0.001 mg/mL (Figure 6A).

An increased production of the enzyme also resulted in iDP cell exposure to 10 µM minoxidil. However, the sample did not stimulate alkaline phosphatase production by primary cells. Similar conclusions were drawn by other authors. Lee et al. [60] analyzed the effect of valproic acid and minoxidil on primary human dermal papilla cells. The authors have concluded that valproic acid and not minoxidil increased the alkaline phosphatase production level by the tested cells. The observed stimulation of enzyme synthesis was caused by the up-regulation of the Wnt/β-catenin pathway. The relation between different Wnt isoforms and an increased production of alkaline phosphatase has been reported previously [61,62]. In a study by Lee et al., minoxidil did not affect the Wnt/β-catenin signaling pathway [60]. The positive effects of minoxidil action on the hair cycle are related to the ERK and protein kinase B (Akt) pathways [63]. Kwack et al. [64] compared the biological activity of five immortalized human scalp dermal papilla cell lines to the primary dermal papilla cells (male origin). It was found that alkaline phosphatase was expressed in KNU201 immortalized cells as well as in the early passage of male dermal papilla cells (p2). Primary cells at passage 6 did not produce alkaline phosphatase. The immortalized cell line KNU201 as well as the cell line used in our study was established using a simian virus 40T (SV40T) and the human telomerase reverse transcriptase (hTERT) gene.

Female- and male-derived dermal papilla cell lines produced a significantly higher concentration of the tested enzyme when treated with L-fucose and chondroitin sulphate disaccharide (Figure 6B,C). These results were observed for all tested concentrations of samples after 72, 120, and 168 h of incubation. An increased enzyme activity was observed with increasing incubation time.

**Figure 7:**
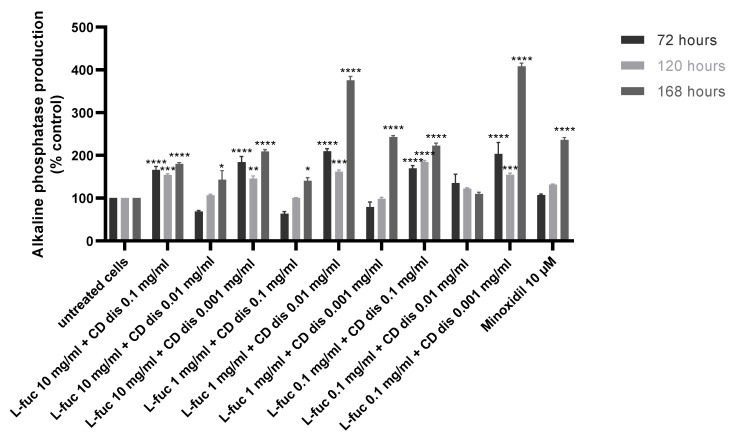
Effect of simultaneous treatment with L-fucose and chondroitin disaccharide Δdi-4S sodium salt on alkaline phosphatase production by immortalized dermal papilla cells. Cells were treated with different concentrations of tested samples for 72, 120, and 168 h. The data are presented as the means ± SEM. Statistical significance in comparison to the negative control (untreated cells) was assessed using a one-way ANOVA followed by Dunnett’s multiple comparison test; * *p* < 0.05; ** *p* < 0.01; *** *p* < 0.001; **** *p* < 0.0001.

The production of alkaline phosphatase by immortalized dermal papilla was stimulated when tested samples were used in combination, especially after 168 h of treatment (Figure 7).

### 2.4. Effect of L-fucose and Chondroitin Disaccharide ∆di-4S Sodium Salt on Glycosaminoglycan (GAG) Production in Dermal Papilla Cells

The extracellular matrix of the hair follicle dermal papilla is rich in glycosaminoglycans, with the expression varying during the hair growth cycle and being maximal in the growth phase (anagen). The ability of the tested samples to support the anagen phase of hair growth was tested with an assessment of glycosaminoglycan production by the three dermal papilla cell lines.

**Figure 8:**
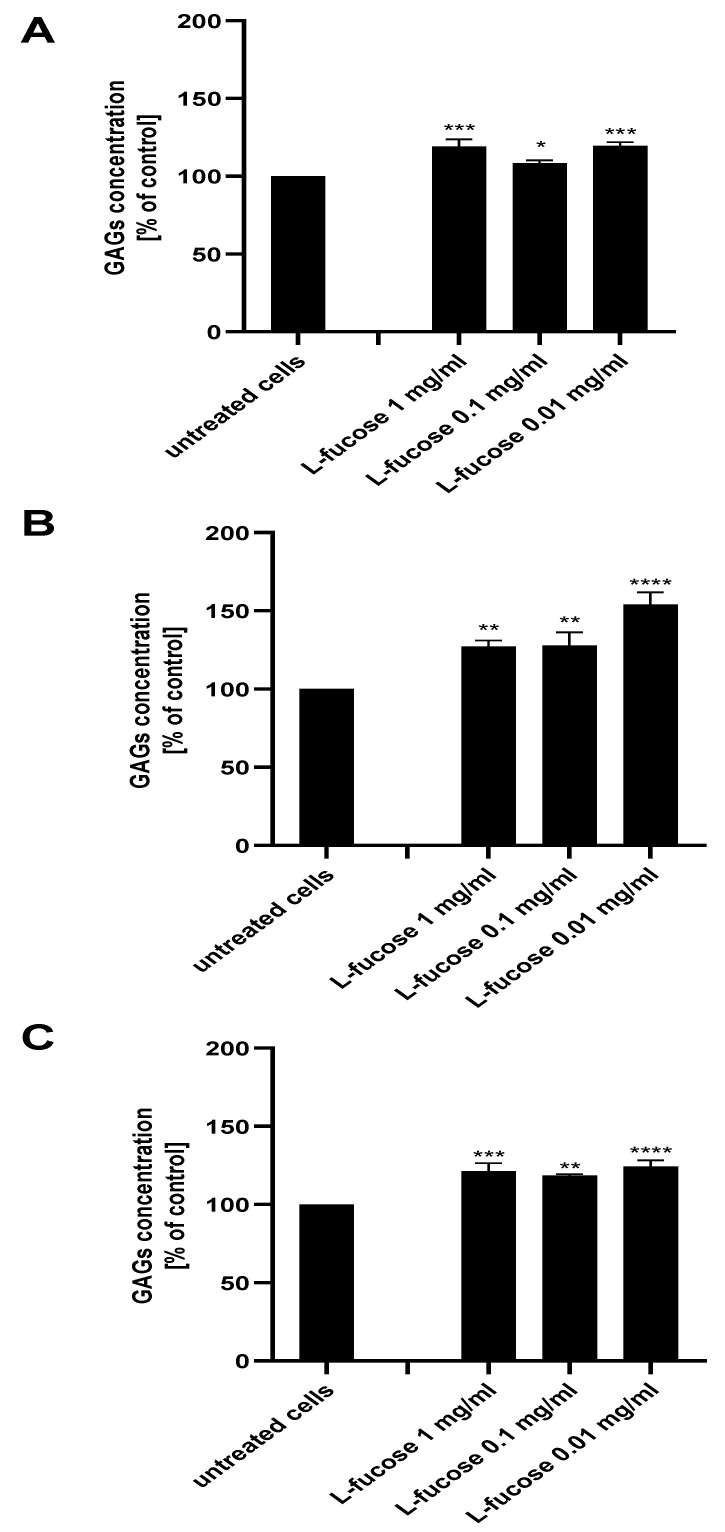
Effect of L-fucose on glycosaminoglycan production by dermal papilla cells: immortalized (**A**), primary male (**B**), and primary female (**C**). Cells were treated with different concentrations of the tested sample for 96 h. The data are presented as the means ± SEM. Statistical significance in comparison to the negative control (untreated cells) was assessed using a one-way ANOVA followed by Dunnett’s multiple comparison test; * *p* < 0.05; ** *p* < 0.01; *** *p* < 0.001; **** *p* < 0.0001.

As can be seen in Figure 8, treatment of all three types of dermal papilla cell lines resulted in a statistically significant increase in glycosaminoglycan production by the cells. The highest expression of sulphated GAGs was obtained for male HFDPC, the levels of detected molecules were 127%, 128%, and 154% for concentrations of 1 mg/mL, 0.1 mg/mL, and 0.01 mg/mL, respectively.

### 2.5. Effect of L-fucose and Chondroitin Disaccharide ∆di-4S Sodium Salt on Collagen Synthesis in Fibroblasts

**Figure 9:**
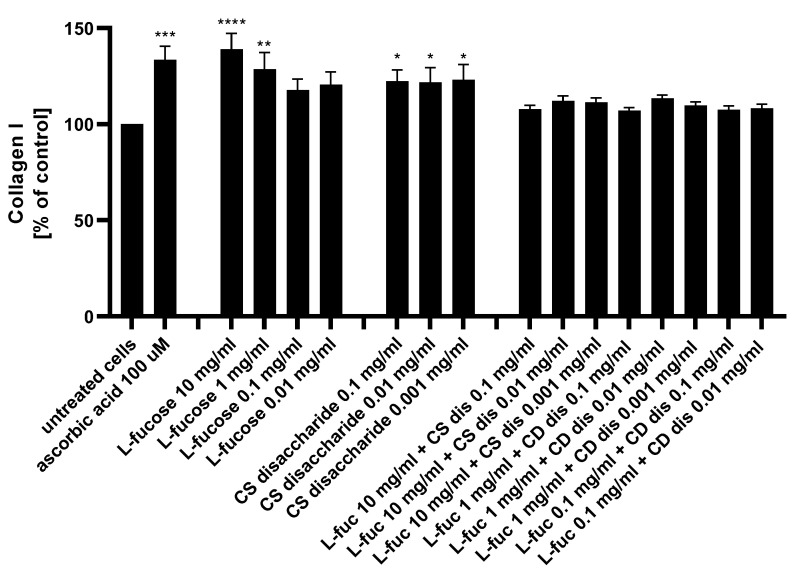
Collagen I production measured by ELISA in HDFa after treatment with L-fucose and chondroitin disaccharide Δdi-4S sodium salt. HDFa was treated with different concentrations of the tested samples for 48 h. Results are presented as a percentage of the untreated cells ± SEM. Statistical significance in comparison to the negative control (untreated cells) was assessed using a one-way ANOVA followed by Dunnett’s multiple comparison test; * *p* < 0.05; ** *p* < 0.01; *** *p* < 0.001; **** *p* < 0.0001.

The potential of the tested samples to stimulate collagen I production by dermal fibroblasts was assessed using the ELISA technique. The results are presented in Figure 9.

L-fucose caused a statistically significant increase in collagen production when used at the two highest concentrations, 10 mg/mL and 1 mg/mL. Human dermal fibroblasts treated with three concentrations of chondroitin sulphate disaccharide Δdi-4S exhibited a statistically significant increase in collagen production levels. There was a lack of significant changes in the protein expression observed when cells were exposed to the combination of both tested samples. Collagen production levels were reduced below those achieved by either L-fucose and chondroitin sulphate disaccharide alone. When L-fucose was used alone at a concentration of 10 mg/mL, the collagen production level was significantly higher when compared to the protein expression obtained after simultaneous treatment with the same concentration of L-fucose and 0.1 mg/mL (*p* < 0.01), 0.01 mg/mL (*p* < 0.05), and 0.001 mg/mL (*p* < 0.05) chondroitin sulphate disaccharide.

### 2.6. Effect of L-fucose and Chondroitin Disaccharide ∆di-4S Sodium Salt on Elastin Synthesis in Fibroblasts

The ability of L-fucose and chondroitin sulphate disaccharide Δdi-4S to stimulate fibroblasts to produce one of the major components of the extracellular matrix, elastin, was analyzed using ELISA.

**Figure 10:**
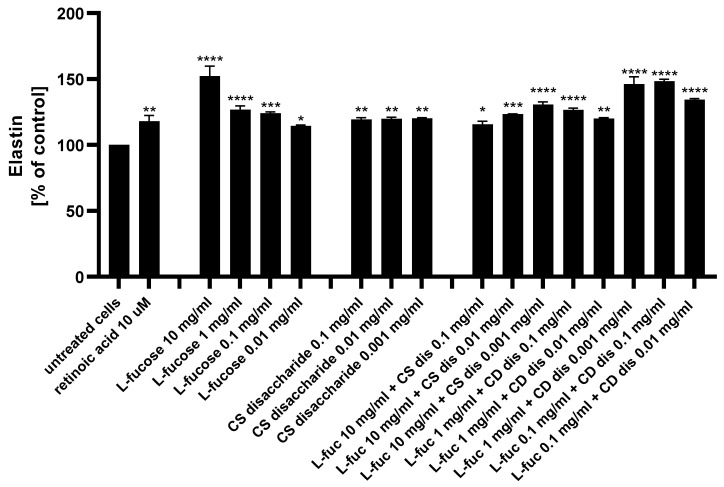
Elastin production measured by ELISA in HDFa after treatment with L-fucose and chondroitin disaccharide Δdi-4S sodium salt. HDFa was treated with different concentrations of the tested samples for 24 h. Results are presented as a percentage of the untreated cells ± SEM. Statistical significance in comparison to the negative control (untreated cells) was assessed using a one-way ANOVA followed by Dunnett’s multiple comparison test; * *p* < 0.05; ** *p* < 0.01; *** *p* < 0.001; **** *p* < 0.0001.

As presented in Figure 10, both tested samples, used alone or in combination, exhibited a statistically significant increase in elastin production by human dermal fibroblasts. Of the 15 treatments evaluated, 4 caused a significantly higher increase in the protein expression when compared to the positive control: 10 µM retinoic acid (*p* < 0.01)–10 mg/mL L-fucose; 1 mg/mL L-fucose + 0.001 mg/mL CS dis; 0.1 mg/mL L-fucose + 0.1 mg/mL CS dis; and 0.1 mg/mL L-fucose + 0.001 mg/mL CS dis. When L-fucose was used alone at a concentration of 10 mg/mL, the elastin expression level was significantly higher compared to the protein production obtained after simultaneous treatment with the same concentration of L-fucose and 0.1 mg/mL (*p* < 0.0001), 0.01 mg/mL (*p* < 0.001), and 0.001 mg/mL (*p* < 0.01) chondroitin sulphate disaccharide. A combination of 0.1 mg/mL L-fucose and 0.1 mg/mL chondroitin sulphate disaccharide resulted in a significantly higher elastin production than that achieved by either L-fucose or chondroitin sulphate disaccharide alone.

### 2.7. Effect of L-fucose and Chondroitin Disaccharide ∆di-4S Sodium Salt on Gene Expression

**Figure 11:**
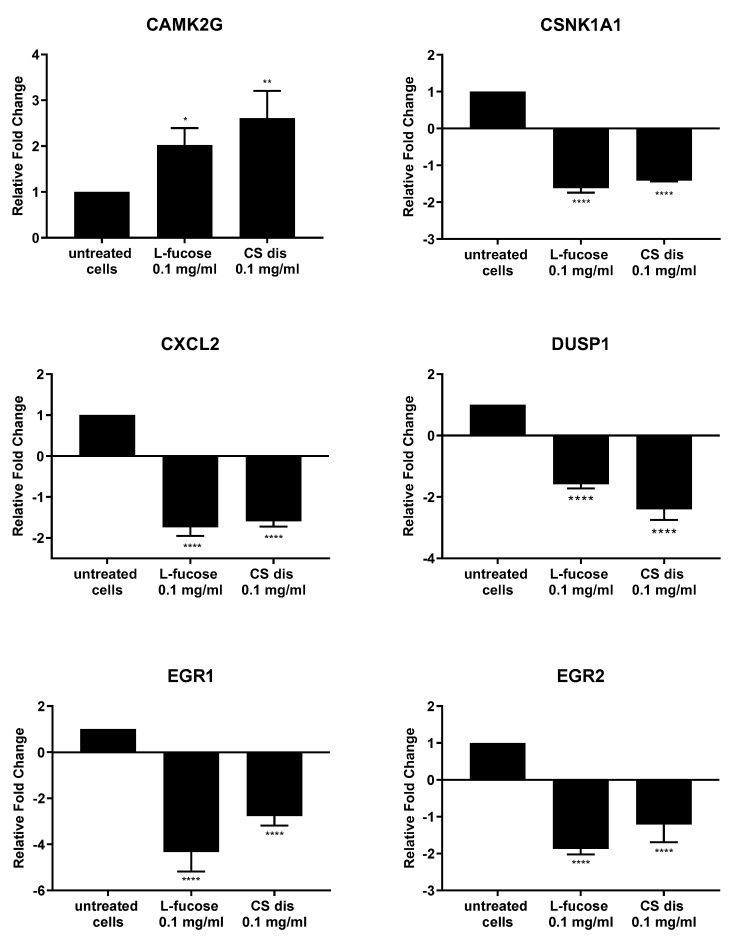

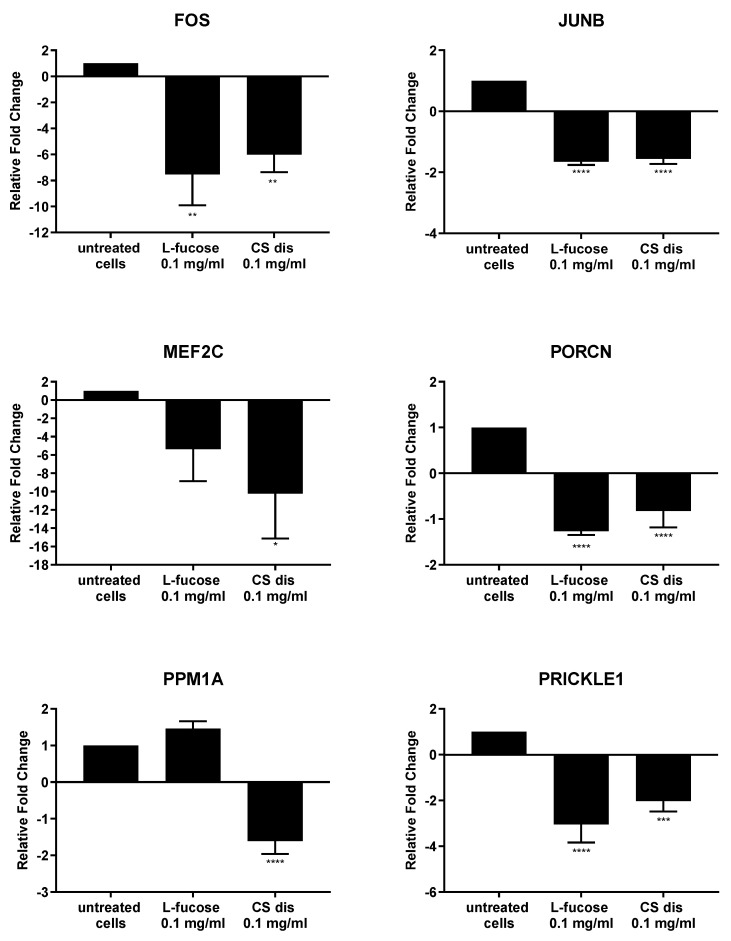
l-fucose’s and chondroitin disaccharide Δdi-4S sodium salt’s effect on gene expression in male human dermal papilla cells. The data are presented as the means ± SEM. Statistical significance in comparison to the negative control (untreated cells) was assessed using a one-way ANOVA followed by Dunnett’s multiple comparison test; * *p* < 0.05; ** *p* < 0.01; *** *p* < 0.001; **** *p* < 0.0001.

As can be seen from Figure 11, both tested samples, L-fucose and chondroitin sulphate disaccharide, caused similar changes in the genetic level. The only exception is the PPM1A gene, the expression of which was significantly down-regulated by CS disaccharide and up-regulated (lack of statistical significance) by L-fucose. In general, treatment of male dermal papilla cells with L-fucose caused an up-regulation of the expression of two genes (CAMK2G and PPM1A) and down-regulation of ten (CSNK1A1; CXCL2; DUSP1; EGR1; EGR2; FOS; JUNB, MEF2C; PORCN; and PRICKLE1). Chondroitin sulphate disaccharide at a concentration of 0.1 mg/mL up-regulated the expression of one gene of interest, CAMK2G, and down-regulated the expression of all other eleven genes.

## 3. Discussion

In the present study, the effect of L-fucose and chondroitin disaccharide Δdi-4S sodium salt on skin and hair cells was investigated. Both compounds are building blocks of larger molecules with proven skin and hair health promoting properties, fucoidan and glycosaminoglycan chondroitin sulphate. Prior work has documented the potential of both tested compounds to support skin and hair health, however, to the best of authors knowledge, the assessment of bioactivity of the samples, used separately and in combination, in four human cell lines, both immortalized and primary, was not considered previously in the literature.

Proliferative properties of tested L-fucose and chondroitin sulphate disaccharide were analyzed in this study. The obtained results indicate that tested compounds positively affect proliferation rate of all four types of tested cell lines. This is highly valued in the context of approach for skin rejuvenation, anti-aging and hair growth. Dermal fibroblast proliferation and skin turnover are associated with and characteristic of both intrinsic and extrinsic skin aging pathways. 

Cellular proliferation is a very important parameter of skin and hair regeneration and is evaluated for every molecule that potentially play a role in hair and skin biology. Proliferation is the most often tested marker of activity of dermal papilla cells, as it determines their growth and mitotic index [65]. Dermal fibroblasts proliferation is not only a parameter considered for the antiaging agents, but also plays a role in wound healing and skin regeneration [66]. Neri et al. [58] investigated hair growth promoting effect of GAGs extracted from ascidian tunics. 

After separation and purification steps, researchers obtained three fractions differing in the ratio of ∆di-6S and ∆di-4S disaccharide components. It was found that none of the tested fraction showed toxicity on human follicle dermal papilla cells and fractions F3 used at the concentrations 50 and 100 μg/mL significantly increased cell proliferation. Proliferative effect was also observed for a combination of fractions F2 and F3 after 120 hours of incubation. The results obtained in our study prove proliferative effect of chondroitin sulphate disaccharide Δdi-4S sodium salt as well. As proteoglycans and building them glycosaminoglycans are present in dermal papilla cells in large quantities, especially in the phase of hair growth, anagen, the mechanism of action of the tested compound might be based on the delivery of building units for these larger molecules [67,68]. Glycosaminoglycans and proteoglycans are important components of not only hair follicles, but skin extracellular matrix as well. The most abundant skin proteoglycans- versican, decorin, and biglycan, belong to the class of chondroitin sulphate or dermatan sulphate proteoglycans [69]. The observed proliferative properties of tested disaccharides on human dermal fibroblasts might be connected to the fact that tested compound is a building unit for skin proteoglycans, molecules involve in the regulation of cell adhesion, migration and proliferation [70]. Another tested compound, L-fucose also showed proliferative activity in dermal papilla cells and dermal fibroblasts. Similar results were obtained by Péterszegi et al. [71]. L-fucose used at concentrations 1 and 10 µg/mL statistically significant increased proliferation of human skin fibroblasts. 

Dermal papilla cells produce a huge variety of growth factors, cytokines and other molecules acted as a marker of hair growth. One of these secreted molecules is alkaline phosphatase [65]. This zinc-metallo enzyme (EC 3.1.3.1) is widely distributed in high metabolic rate cells [72]. Hair follicles as a highly proliferating and regenerative tissue also express substantial amount of alkaline phosphatase. The activity of the enzyme changed during the hair growth cycle—with a maximal level obtained during anagen phase of hair growth [73]. On that basis it can be assumed that the enzyme act as a marker of the hair growth phase. Results of present in vitro study on dermal papilla cells revealed that tested L-fucose and chondroitin sulphate Δdi-4S disaccharide promote expression of alkaline phosphatase and through that activity support the anagen phase of hair cycle. The increase in the enzyme level after treatment with potential hair growth promoters was observed and documented previously in the literature. Treatment of human dermal papilla cells with rice bran mineral extract resulted in increase in enzyme production. The hair growth promoting activity of tested sample was connected with the activation of β-catenin/Wnt signaling pathway - one of the most important for growth and development of hair follicles pathway [74]. The Wnt signaling pathway is an evolutionarily conserved pathway regulating cell migration, cell polarity, neural patterning and organogenesis during embryonic development [75]. Within the group of Wnt pathways we can distinguish canonical (Wnt-β-catenin) and noncanonical signalling (the Wnt/Ca2+, the Wnt/planar cell polarity (PCP) and Wnt/Ror2) [76,77] and all are involved in hair follicle development [63,78,79,80,81]. 

Male follicle dermal papilla cells treatment with L-fucose and chondroitin sulphate disaccharide resulted in increased expression of CAMK2G (Figure 11), gene involves in Wnt/Ca2+ pathway. Calmodulin-dependent kinase II (CamKII), product of the gene expression, is stimulated by calcium released from the endoplasmic reticulum and activates transcription factor NFAT in noncanonical Wnt pathway [82]. PRICKLE1, another gene from the Wnt noncanonical signalling pathway, was found to be significantly downregulated by the tested compounds. The gene encodes prickle planar cell polarity protein 1, a nuclear receptor involve in the Wnt/PCP pathway. Several studies indicate that PRICKLE1 may function as a negative regulator of canonical Wnt/β-catenin pathway [83,84,85,86]. Moreover, the significant upregulation of CSNK1A1 was observed when primary cells were treated with tested samples. Protein encoded by the gene, casein kinase 1α (CK1α), is a component of the β-catenin degradation complex and acts as a critical regulator of the Wnt signaling pathway [87,88,89]. Casein kinase 1α in responsible for β-catenin phosphorylation, which initiates cascade of signals leading to the β-catenin destabilization, ubiquitination and subsequently proteasomal degradation [90]. The obtained RT-PCR results may indicate that the activity of L-fucose and chondroitin sulphate disaccharide is mediated through Wnt signalling pathways. 

Downregulation of two genes, FOS and JUNB, which protein products are the members of activator protein 1 (AP-1) family of transcription factors was observed in the present study. AP1 proteins are responsible for gene expression regulation and are involved in a variety of cellular processes, like proliferation, differentiation or apoptosis [91,92,93,94]. One of the signalling pathway AP-1 proteins are involved in is TGF-β-Smad. It was observed that knockdown of two subunits of AP-1, JUN and FOS or using inhibitors, T-5224 and SR11302, resulted in downregulation of TGF-β1 [95]. TGF- β acts as a negative regulator of hair growth, reduces the anagen phase and promotes early enter the catagen [96,97]. The synthesis of transforming growth factor-beta2 in dermal papilla cells as a result of dihydrotestosterone activity is an initial step of a cascade of molecular events leading to the premature entry into catagen [97]. The results obtained in the study may suggest that hair growth properties of tested samples are connected with the inhibition of TGF-β pathway, involved in the anagen reduction and catagen progression.

In the present study, the effect of L-fucose on glycosaminoglycans production by dermal papilla cells was investigated. Results showed increase in GAGs level after treatment with tested sample. Positive effect of L-fucose activity was observed in all three types of dermal papilla cells, both immortalized and primary male- and female-derived. L-fucose is an element of many glycoproteins as a terminal or core monosaccharide of glycan chain, can be found in proteoglycans like keratan sulphate or chondroitin sulphate. Due to the terminal location of the L-fucose, it might act as a recognition site for other molecules or acceptor molecule for the attachment of further saccharides [98]. It was proven that the dermal part of hair follicle, consisting of dermal papilla and connective tissue sheath, is rich in extracellular matrix compounds, such as glycosaminoglycans (GAGs) [99,100,101,102]. 

The role of these molecules and associated proteoglycans is stabilizing, participating in fibrillogenesis and epithelial-stromal interactions during hair follicle morphogenesis, interacting with secreted growth factors and modulating the signal molecules exchange between the epithelium and the dermal papilla [68,103]. It was reported by Fernández-Martos et al. [104] that a defined GAG matrix promoted and supported the sustained growth of human hair follicles ex vivo. Hair follicular units, obtained from human scalp samples, were grown in supplemented Williams E medium containing 1.5% HC007 containing GAGs (high and low molecular weight hyaluronic acid chondroitin sulfate, dermatan sulfate, keratan sulfate and heparan sulfate, purity ≥ 95%). It was found that the treatment supported cell proliferation and hair follicle growth—after 8 days of ex vivo culture strong cell proliferation phenotype was maintained, after 16 days a significant increase in the number of cells was observed. Moreover, researchers noted increase in hair shaft length in about 50% of hair follicles grown in 1.5% HC007. 

A decrease in skin hydration, loss of compounds that are components of extracellular matrix, such as hyaluronic acid, collagen or elastin, increased activity of matrix-degrading metalloproteinases are associated with the aging process and are visible on the skin in the form of wrinkles or loss of firmness [2,105]. The dermis consists mainly of collagen fibers, which make up 70% of its volume. The most abundant collagen in the skin is type I collagen. It is a fibrous structural protein with significant tensile strength giving the skin its strength and toughness [106]. Collagen is produced by dermal cells, fibroblasts. This type of cells is responsible for production the other fibrillar skin protein, elastin. This component of extracellular matrix provides skin with its stretch and resilience, exemplified by the skins ability to return to their original conformation [107]. Compounds that have an ability to stimulate fibroblasts to produce extracellular matrix molecules - collagen and elastin, and thus compensate for the aging resulting changes, are considered to be an anti-aging agents. In the present study, increased synthesis of collagen and elastin was observed as a result of dermal fibroblasts treatment with L-fucose and chondroitin sulphate disaccharide (Figure 9 and Figure 10). Robert et al. [108] investigated the effect of L-fucose on elastin biosynthesis on human skin fibroblasts in explant cultures. L-fucose used at concentration 10 µg/mL causes increase in elastin production of about 40%. It was also found that 10 µg/mL L-fucose inhibited collagen biosynthesis. The obtained results are in agreement with our finding. All four tested concentrations of L-fucose used in our study caused statistically significance increase in elastin level, however in the case of collagen the significant increase was observed only for the highest two concentrations, 1% w/v and 0.1% w/v (corresponding to 10 mg/mL and 1 mg/mL). Kitazawa et al. [109] analysed effect of low molecular weight chondroitin sulphate, high molecular weight chondroitin sulphate and chondroitin sulphate disaccharides on ECM molecules production by normal human dermal fibroblasts. The cells were treated with tested samples and the gene expression levels of extracellular matrix-related proteins after 12 h was evaluated. Disaccharide CS significantly increased gene expression levels of COL1A1 and DCN, ELN and SMAD2 when compared with the normal group. The effect of high-molecular-weight CS on gene expression levels was not observed. Significant increase in collagen and elastin protein expression level measured by ELISA was noted as well. 

The obtained results reveal that both tested compounds, L-fucose and chondroitin sulphate disaccharide, support skin and hair health, especially in terms of anti-aging effects. Treatment with tested samples provides the approaches to addressing both intrinsic and extrinsic aging of skin and hair. Tested compounds support and promote proliferation of dermal fibroblast and dermal papilla cells, provide cells with a supply of sulphated disaccharide GAG building blocks, increase ECM molecules production, collagen and elastin, by HDFa, support growth phase of hair cycle, anagen. 

## 4. Materials and Methods

### 4.1. Materials

L-fucose (F2252; ≥99%) and chondroitin disaccharide Δdi-4S sodium salt (C4045) were purchased from Sigma-Aldrich Co. Ltd. (Saint Louis, MO, USA).

### 4.2. Cells Culture

The human dermal fibroblasts cell line, HDFa, was purchased from Gibco^®^ Cell Culture. Cells were cultured in complete Dulbecco’s modified Eagle’s medium (DMEM; Gibco^TM^, Thermo Fisher Scientific, Waltham, MA, USA) containing 5% fetal bovine serum (FBS; Gibco^TM^, Thermo Fisher Scientific, Waltham, MA, USA) and 2 mM L-glutamine (Gibco^TM^, Thermo Fisher Scientific, Waltham, MA, USA) at 37 °C in a 5% CO_2_ humidified atmosphere.

The immortalized human dermal papilla cells, iDPC, were purchased from Kyungpook National University (South Korea). Cells were cultured in complete Dulbecco’s modified Eagle’s medium (DMEM; Gibco^TM^, Thermo Fisher Scientific, Waltham, MA, USA) with GlutaMAX™, 10% FBS, and penicillin/streptomycin (100 unit/mL and 100 μg/mL, respectively; Gibco^TM^, Thermo Fisher Scientific, Waltham, MA, USA).

### 4.3. Proliferation Assay of Dermal Papilla Cells

Dermal papilla cells were seeded at a density of 10,000 cells per well in 96-well plates in 100 μL of complete DMEM. Cells were allowed to adhere for 24 h. Treatment media were prepared to contain an appropriate concentration of L-fucose and chondroitin disaccharide Δdi-4S sodium salt in complete cell culture media. Treatments were performed in triplicate wells. Cells were cultured for a further 24, 48, and 72 h. No media changes were performed between initial treatment and cell assays. Only the medium was used to treat controls cells. After exposure for the desired period of time, 11 µL of PrestoBlue reagent was added to each well of the 96-well plate. Plates were incubated in the dark for 2 h at 37 °C. The fluorescence was read using a 560 nm excitation/590 nm emission filter set (10 nm bandwidth). Fluorescence data in wells containing cells were corrected for background fluorescence using cell-free media control replicates.

### 4.4. Proliferation Assay of Human Dermal Fibroblasts

Human dermal fibroblasts (HDFa) were seeded at a density of 5000 cells per well in 96-well plates in 100 μL complete medium. Cells were allowed to adhere overnight. Treatment media were prepared to contain an appropriate concentration of L-fucose and chondroitin disaccharide Δdi-4S sodium salt in complete cell culture media. Treatments were performed in triplicate wells. Cells were cultured for a further 24, 48, and 72 h. Only the medium was used to treat controls cells. After exposure for the desired period of time, 11 µL PrestoBlue reagent was added to each well of the 96-well plate. Plates were incubated in the dark for 2 h at 37 °C. The fluorescence was read using a 560 nm excitation/590 nm emission filter set (10 nm bandwidth).

### 4.5. Measurement of Alkaline Phosphatase Level

The SensoLyte^®^ Alkaline Phosphatase Assay Kit (AnaSpec) was used for the quantitative evaluation of enzyme production by dermal papilla cells. Cells were seeded in transparent 12-well plates at a density of 30,000 cells per well in 2 mL of complete media. Cells were allowed to adhere overnight. The treatment medium was prepared to contain an appropriate concentration of L-fucose and chondroitin disaccharide Δdi-4S sodium salt in complete DMEM. Cells were assessed for alkaline phosphatase expression at 72, 120, and 168 h. Cells were gently washed twice with 1X assay buffer. Lysis buffer (1X assay buffer with TritonX-100) was added to each well. Cells were transferred into Eppendorf tubes and subjected to three freeze–thaw cycles to ensure cells lysis. Cell suspensions were centrifuged at 2500× *g* for 10 min at 4 °C. The supernatants were used in an AP assay according to the manufacturer’s protocol.

### 4.6. Histological Analysis of Alkaline Phosphatase

Dermal papilla cells were seeded at a density of 10,000 cells in each 8 Cell Culture Chamber FlexiPERM^®^ (Sarstedt AG & Co., Nümbrecht, Germany) on a microscope slide. Cells were allowed to grow for 24 h and were incubated with appropriate concentrations of L-fucose and chondroitin disaccharide Δdi-4S sodium salt for 120 h. A BCIP/NBT tablet (SigmaFastTM BCIP-NBT; Sigma-Aldrich, Saint Louis, MO, USA) was dissolved in 10 mL distilled water to prepare the substrate solution. Cells were washed with Wash Buffer (Tween20 in PBS) without disrupting the cell monolayer. After liquid aspiration, cells were covered to a depth of 2–3 mm with ice-cold 100% methanol and were allowed to fix for 15 min at −20 °C. Cells were then washed with Wash Buffer. The BCIP/NBT substrate solution was added to cover the cellular monolayer. The slides were incubated at room temperature in the dark for 10 min. The substrate solution was aspirated and the cell monolayer was washed with Wash Buffer. Cells were analyzed under a light microscope.

### 4.7. Measurement of GAGs Level

A Blyscan™ Glycosaminoglycan assay, a quantitative dye-binding method, was used for the analysis of sulphated glycosaminoglycans. Dermal papilla cells were seeded at a density of 200,000 cells in T25 flasks containing 4 mL of complete media. Cells were allowed to adhere overnight. Treatment media were prepared to contain an appropriate concentration of stimulants, L-fucose, and chondroitin disaccharide Δdi-4S sodium salt. Media were removed from all T25 flasks and retained for the sGAG assay. Cells were washed with PBS, drained, and 2 mL of Papain Extraction Reagent was added to each flask. Cells were incubated with Papain Extraction reagent at 65 °C with occasional mixing. After 3 h, the digested extracts were removed from the flasks and centrifuged at 10,000 rpm for 10 min. An amount of 50 μL of each supernatant was retained for the sGAG assay according to the manufacturer’s protocol. The sGAG concentration was obtained from the standard curve.

### 4.8. Measurement of Collagen Synthesis

Human dermal fibroblasts were seeded in 6-well plates at a density of 200,000 cells/well. Treatment media were prepared to contain an appropriate concentration of L-fucose and chondroitin disaccharide Δdi-4S sodium salt in DMEM with 1% of FBS. Cells were incubated with the tested compounds for 120 h. The synthesis of type I collagen by human dermal fibroblasts was determined using a commercially available enzyme-linked immunosorbent assay (R&D Systems, Minneapolis, MN, USA) according to the manufacturer’s protocol.

### 4.9. Measurement of Elastin Synthesis

Human dermal fibroblasts were seeded in 6-well plates at a density of 200,000 cells/well. Treatment media were prepared to contain an appropriate concentration of L-fucose and chondroitin disaccharide Δdi-4S sodium salt. Cells were incubated with the tested compounds for 120 h. The synthesis of elastin by human dermal fibroblasts was determined using a commercially available enzyme-linked immunosorbent assay (BT-Labs, Birmingham, UK) according to the manufacturer’s protocol.

### 4.10. RNA Extraction and Quantitative RT-PCR

Total RNA was extracted from the male dermal papilla cells using a High Pure RNA Isolation kit (Roche) according to the manufacturer’s protocol. A total of 500 ng of DNA-free RNA was subjected to reverse transcription using a qScript cDNA Synthesis Kit (Quanta bio, Beverly, MA, USA). The relative cDNA levels were measured using FastStart Essential DNA Green Master (Roche) in a LightCycler^®^ 96 System (Roche). Two housekeeping genes, GAPDH (glyceraldehyde-3-phosphate dehydrogenase) and ACTB (actin beta), were used as reference genes in the calculations of relative expressions. The relative mRNA expression level was calculated using the 2^−ΔΔCT^ analysis method. All measurements were repeated in three biological and three technical replicates. The primers used for the quantitative PCR are shown in Table 1.

### 4.11. Statistical Analysis

All results were expressed as the means ± SEM from three independent experiments. GraphPad Prism 9 (GraphPad Software, San Diego, CA, USA) and Dunnets’s multiple comparison tests were used to analyze significant differences (*p* < 0.05) between the mean values of the individual group.

## Figures and Tables

**Table 1:** Sequences of primer pairs used in RT-PCR.

Gene Name	Forward Primer Sequence (5′ → 3′)	Reverse Primer Sequence (5′ → 3′)
**GAPDH**	TCGACAGTCAGCCGCATCTT	GCCCAATACGACCAAATCCGT
**ACTB**	GAGCACAGAGCCTCGCCTTT	CATCACGCCCTGGTGCCT
**CAMK2G**	CCCGTCTCCTCCTCTTGCTC	ACAGAGAAAGCACCCTTGCC
**CSNK1A1**	CTCTTCCCAGAGGTGTCGGG	GCTTCACTGCCACTTCCTCG
**CXCL2**	AGGGGTTCGCCGTTCTCGG	CGAGGAGGAGAGCTGGCAAGG
**DUSP1**	CACTCTACGATCAGGGTGGC	TCCTTGCGGGAAGCGTGATA
**EGR1**	TGACCGCAGAGTCTTTTCCTG	CCAGGGAAAAGCGGCCAGTA
**EGR2**	GCGAGGAGCAAATGATGACCG	TTGATCATGCCATCTCCGGC
**FOS**	CACTCCAAGCGGAGACAGACC	AGGCCCCCAGTCAGATCAAG
**JUNB**	CGCATCAAAGTGGAGCGCAA	TTCTCGGCCTTGAGCGTCTT
**MEF2C**	AGTGCAGGGAACGGGTATGG	GCAGGTCGACATCCTCAGACA
**PORCN**	CTTCGCAAGTGGCTGCGAG	TCCACCATTGACCGAGGCAG
**PPM1A**	GAGGCGCGAAAGCGATGAG	CAGATCATCCGGGCGTTGGA
**PRICKLE1**	TTCTGGGCTCTGGATGGTTCG	TCAAACAATGGCTGCTCGC

## Data Availability

Data are contained within the article.
